# Inferior Vena Cava Atresia: A Rare Cause of Recurrent Deep Venous Thrombosis and Infected Venous Ulcers

**DOI:** 10.7759/cureus.87729

**Published:** 2025-07-11

**Authors:** Fawad Talat, Saqib Gul, Owais Gul, Maria Khalid

**Affiliations:** 1 Internal Medicine, United Health Services Wilson Medical Center, Johnson City, USA; 2 Internal Medicine, Hamdard College of Medicine & Dentistry, Karachi, PAK

**Keywords:** anticoagulation, dvt, ivc atresia, vascular anomaly, venous ulcers

## Abstract

Inferior vena cava (IVC) atresia is a rare vascular anomaly and may lead to recurrent deep venous thrombosis (DVT), especially in young adults. It leads to venous stasis due to insufficient drainage from the lower extremities, which in turn contributes to the formation of venous ulcers. Imaging modalities such as computed tomography (CT) or magnetic resonance imaging may aid in diagnosis. There are no specific guidelines regarding anticoagulation for DVTs in these patients, so general principles of management are usually followed. Surgical options are less studied due to the low incidence of the condition and are, therefore, less likely to be advised. In this report, we present a case of a 37-year-old male patient presenting with recurrent DVTs and infected venous ulcers with a delayed incidental diagnosis of IVC atresia on CT imaging.

## Introduction

Congenital malformations involving the inferior vena cava (IVC), such as agenesis or atresia, may lead to deep venous thrombosis (DVT), and patients usually present at a young age [1]. DVTs can be bilateral and/or recurrent. IVC atresia is an extremely rare vascular anomaly found in approximately 1% of the general population. Because of its low incidence, studies are limited in clearly depicting the goal and duration of anticoagulation for DVTs among the affected patients. Additionally, there is less data available to support surgical therapeutic options [2]. We present here a case of a young patient who came with a history of recurrent DVTs as well as infected venous ulcers. IVC atresia was diagnosed incidentally on an abdominal CT scan obtained for the patient's nonspecific symptoms.

## Case presentation

A 37-year-old male with a past medical history of recurrent DVTs involving bilateral lower extremities, infected venous ulcers and recurrent cellulitis of bilateral lower legs, poly-substance use disorder, and chronic nicotine dependence presented to the hospital with severe pain in both legs. He was following up with the outpatient wound care clinic for dressing changes for his chronic bilateral lower leg wounds. The patient was informed that his wounds were not healing properly, and hence was recommended to undergo further evaluation at the hospital. He was also following up with the vascular surgery department in the outpatient setting and had undergone venous ablation surgeries twice in the past. He was also prescribed apixaban previously due to his recurrent DVTs for an indefinite duration, but the patient reported non-compliance with anticoagulants. He complained of sharp pain (8/10 on the pain scale) around his ulcer sites that was associated with increased drainage from wounds and redness of bilateral lower legs. The review of systems was positive for fatigue but negative for any fever, chills, nausea, vomiting, headache, chest pain, shortness of breath, and urinary or bowel complaints.

**Figure 1:**
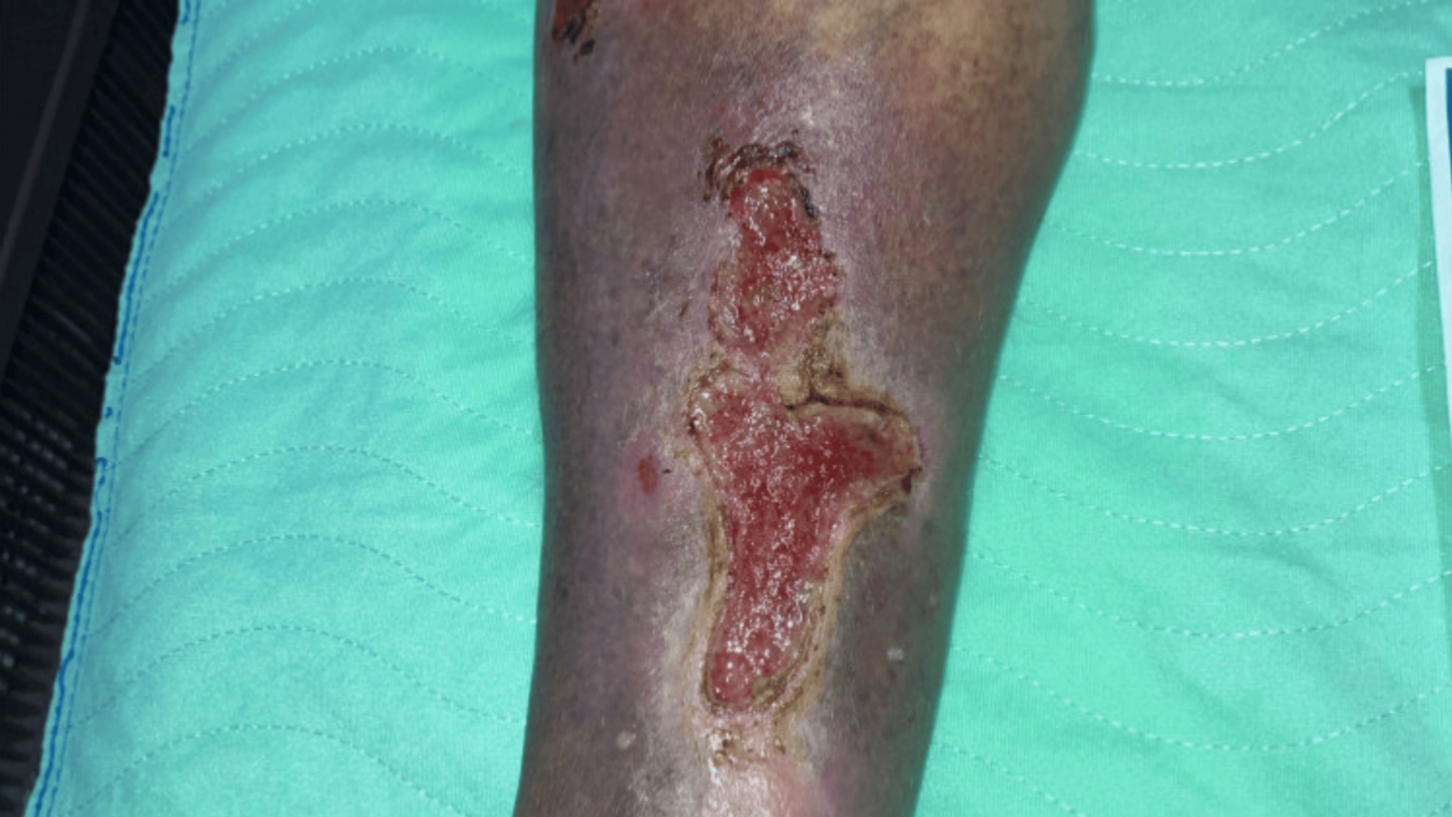
Right leg venous ulcers on examination

On physical examination, he was afebrile; his heart rate and respiratory rate were within normal limits. His blood pressure was 115/71 mmHg, and his oxygen saturation was normal on room air. The patient was alert and oriented on arrival. His chest was clear to auscultation, and his heart sounds were regular with normal S1 and S2. His abdomen was soft, non-distended, and non-tender on palpation. The patient's right leg examination showed at least two wounds, the largest one located medially and measuring up to 8 x 3 cm in length and width (Figure 1).

**Figure 2:**
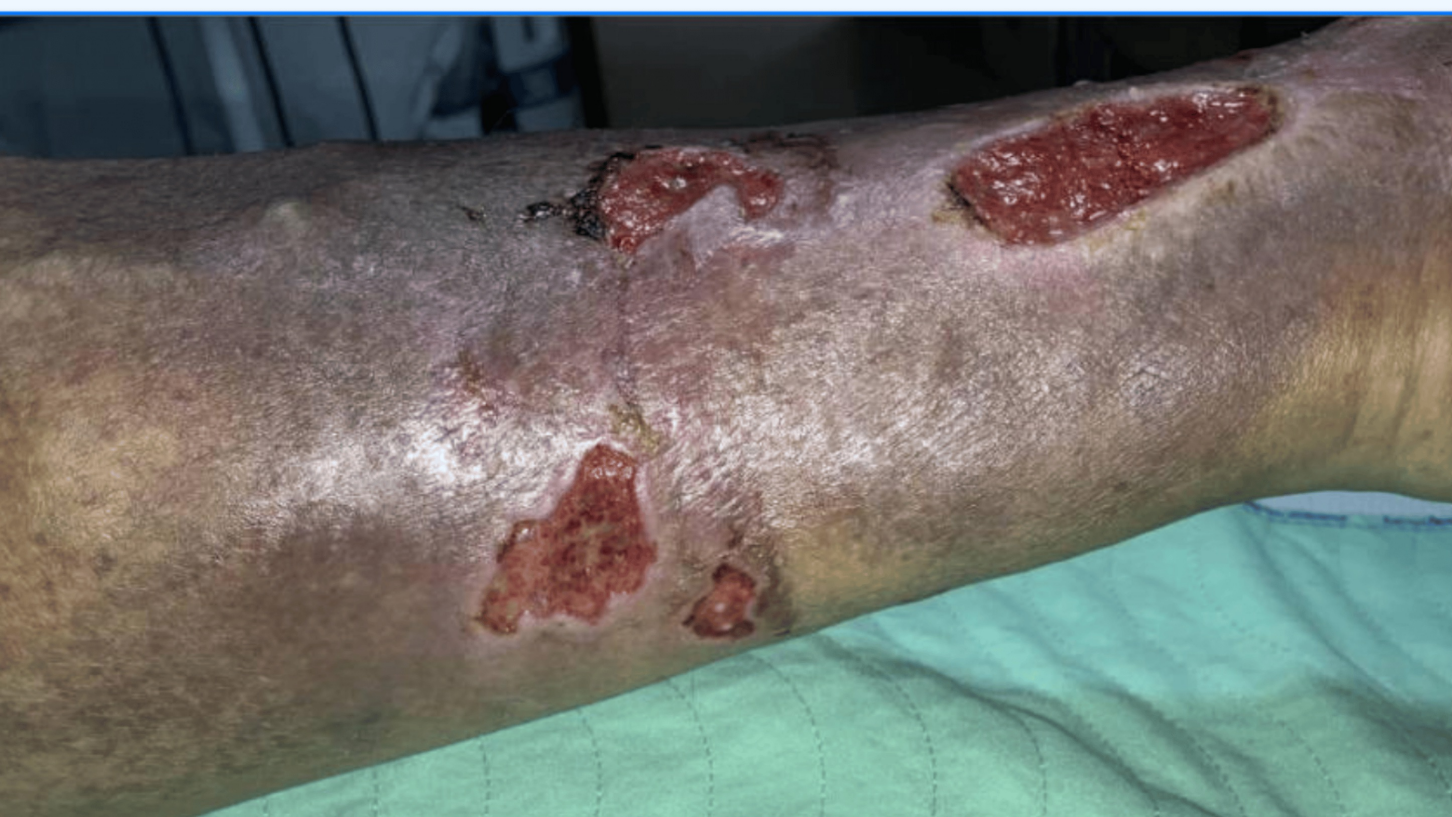
Left leg venous ulcers on examination

His left leg examination showed at least three wounds located in the pre-tibial, medial, and around the left ankle regions. A moderate amount of sero-sanguineous and purulent discharge was present with a foul odor. Areas around these wounds were tender to palpation and somewhat erythematous (Figure 2).

**Figure 3:**
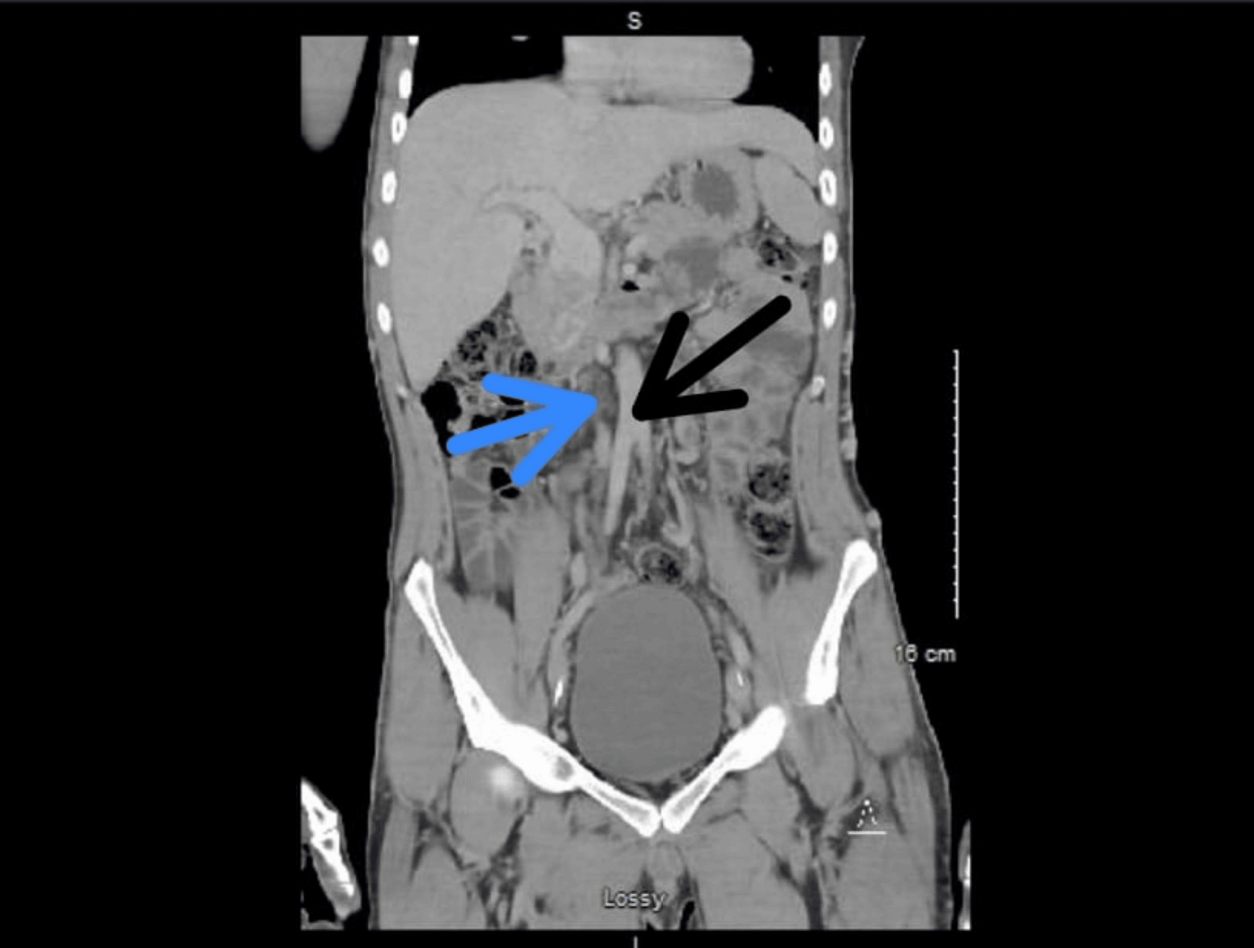
CT scan (coronal view) of the abdomen with contrast showing the area of absent IVC (blue arrow) right next to the normal-appearing abdominal aorta (black arrow) IVC: inferior vena cava

**Figure 4:**
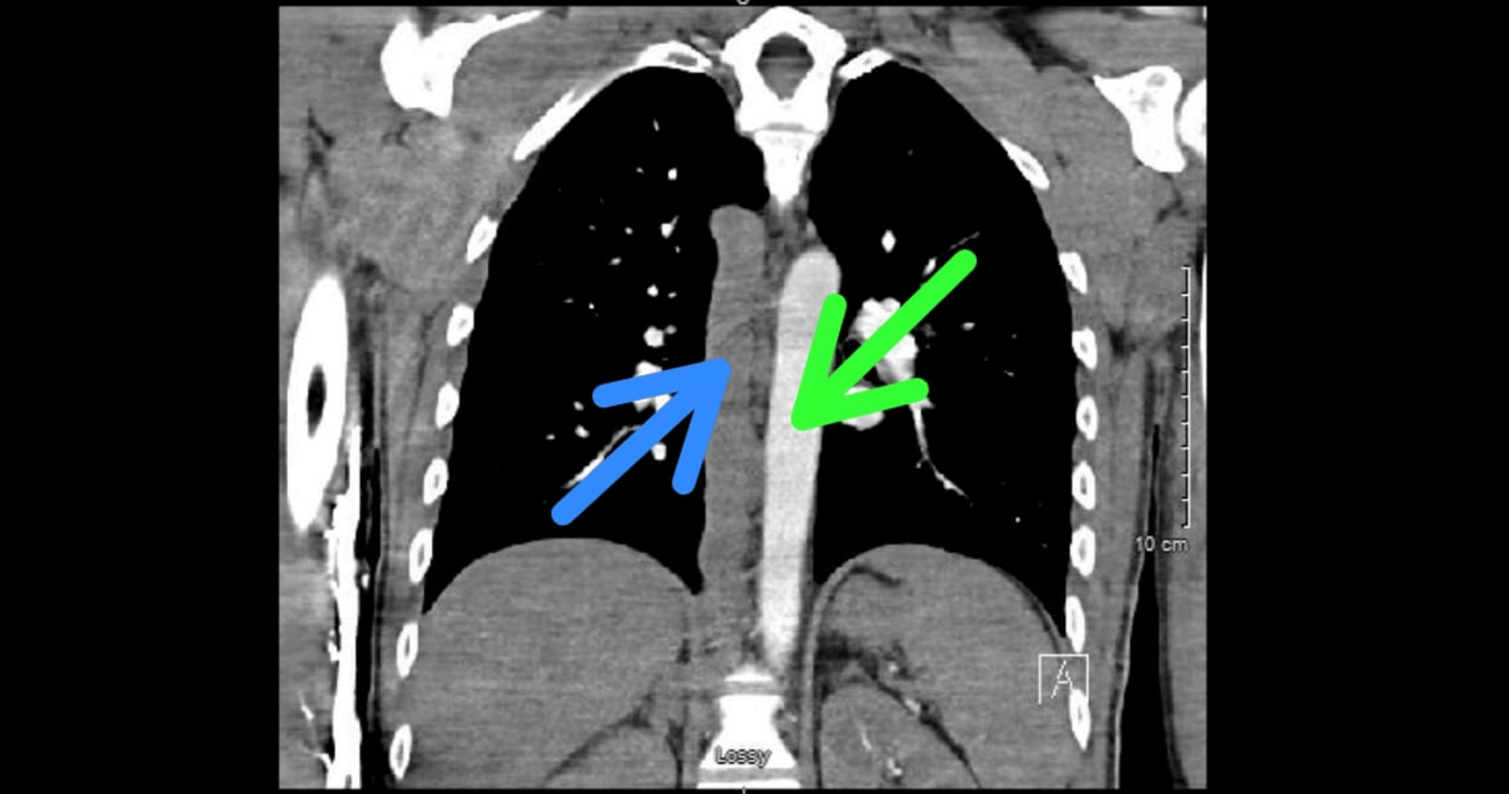
CT angiogram (coronal view) of the chest showing a prominent azygos vein (blue arrow) right next to the normal-appearing thoracic aorta (green arrow)

The laboratory work-up showed a normal white blood cell count of 7.3 x 10^9^/L (normal range: 4.5-10.0 x 10^9^/L) and elevated C-reactive protein (CRP) of 5.3 mg/dL (normal value: <0.9 mg/dL). Two sets of blood cultures were collected, and they both came back negative. Wound cultures obtained from both legs came back positive for methicillin-sensitive *Staphylococcus aureus* (MSSA) as well as *Pseudomonas aeruginosa*. These culture results were similar to those obtained during previous encounters at the wound care clinic, and both organisms were found to be pan-sensitive to the anti-microbials. A computed tomography (CT) scan, without contrast, of bilateral lower extremities was obtained that showed superficial thickness ulcers with adjoining soft tissue stranding that was consistent with cellulitis. Tortuous superficial veins were demonstrated bilaterally. A Doppler venous ultrasound of the bilateral lower extremities was conducted that revealed DVT in one of the two left distal femoral and popliteal veins, which appeared acute. DVTs noted within the left common femoral vein, left proximal through mid-femoral vein, and left deep femoral vein appeared chronic. Chronic appearing DVTs were also found within the right common femoral and right proximal femoral vein. The patient was started on a course of oral ciprofloxacin and doxycycline for seven days for his infected venous ulcers. Apixaban was restarted at a therapeutic dose of 10 mg twice daily for the initial seven days, followed by 5 mg twice daily. He subsequently developed abdominal pain that, according to him, was radiating upwards from the bilateral lower extremities. For this reason, he underwent a CT scan of the abdomen and pelvis with intravenous (IV) contrast that showed patent portal, mesenteric, and splenic veins. However, it demonstrated the presence of extensive varices in the peri-portal region with the questionable absence of IVC (Figure 3). CT angiogram of the chest, done as part of the venous thrombo-embolism workup, ruled out pulmonary embolism but revealed the presence of prominent azygos as well as hemiazygos veins in the lower chest (Figure 4).

The vascular surgery department was consulted after these suspicious imaging findings. However, as per their recommendation, the patient was referred to a higher level care center to undergo evaluation for caval reconstruction surgery.

## Discussion

IVC atresia is a rare vascular anomaly, characterized by either the absence or incomplete development of IVC. It is often the result of aberrancy in the complex embryological process involved in the development of the IVC, which entails the fusion and regression of multiple embryological veins. In some cases, in vitro thrombophilia can act as a precipitating factor for IVC atresia [1]. Studies indicate that IVC atresia is found in almost 5% of patients under the age of 30 with unprovoked lower extremity DVT [2]. Patients with IVC atresia have a 10-fold increased risk of thrombosis. Studies show that patients with DVTs who are diagnosed with IVC atresia are usually in their third decade of life [1]. Our patient also belonged to the same demographic group.

In Virchow's triad, IVC atresia contributes to venous stasis that is the most likely mechanism resulting in DVTs. Venous stasis is a consequence of compromised drainage from the lower extremities and is also responsible for recurrent ulcers seen in patients with IVC atresia. IVC atresia is not frequently associated with pulmonary embolism because patients with IVC atresia develop robust collateral circulation through the azygos and hemiazygos veins. As a result, the emboli tend to get trapped in the azygos/hemiazygos venous system preventing them from reaching the pulmonary circulation. However, it is worth mentioning that collateral circulation is insufficient for lower limb venous drainage, leading to venous stasis [3].

Studies indicate that approximately one-third of patients with IVC atresia have hypercoagulable disorders [3]. One systematic review found that Factor V Leiden deficiency followed by hyperhomocysteinemia were the most common coagulation abnormalities seen in patients with IVC atresia [4]. Iversen et al. also described the association between IVC atresia and antithrombin III deficiency. Therefore, it is also important to screen patients with IVC atresia for coagulation abnormalities. Apart from hypercoagulable disorders, IVC atresia is associated with several other conditions, including transposed abdominal viscera, polysplenia or asplenia, renal hypoplasia or agenesis, and dysgenesis of the lungs [1].

Patients with IVC atresia can present at various stages of life depending on whether the condition is associated with other congenital abnormalities. However, some patients with isolated IVC atresia may remain asymptomatic, and the pathology may be discovered incidentally by imaging done for non-specific reasons. Saab et al. in their systematic review mentioned that leg swelling followed by leg pain were the most common presenting symptoms in patients with IVC atresia. They also found that 64.3% of patients with IVC atresia had presented with DVT. Some patients with this condition might present with nonspecific symptoms such as abdominal pain or back pain [3]. Our patient also presented with recurrent DVTs and underwent imaging for nonspecific complaints such as abdominal pain that led to the diagnosis.

The diagnosis of IVC atresia requires CT or MRI. These imaging studies should be pursued in younger patients with unprovoked DVTs. In clinical practice, Doppler ultrasound and venography are the commonly employed tools for the diagnosis of DVT, but these studies can miss the diagnosis of IVC atresia. Therefore, IVC atresia may very well be underdiagnosed [3]. There are no specific guidelines for the management of DVTs in patients with IVC atresia. For anticoagulation, several small-scale studies have reported the safety and efficacy of direct oral anticoagulants (DOACs) among this particular patient population [5]. With regard to the duration of anticoagulation, some experts suggest lifelong anticoagulation for IVC atresia patients presenting with new-onset DVT while others argue that indefinite anticoagulation for this young population cohort might carry more risks than benefits [3]. However, lifelong anticoagulation is reasonable in patients with recurrent DVTs and IVC atresia. Patients with IVC atresia and iliofemoral DVT have also been successfully treated with catheter-directed thrombolysis. A large number of studies in the literature have shown that catheter-directed thrombolysis results in the resolution of symptoms without a significant risk of post-thrombotic syndrome [6,7]. Reslan et al. concluded in their literature review that the practice of catheter-directed thrombolysis is safe and effective in patients with IVC atresia and DVTs [8].

There is no consensus on the optimal approach for the surgical treatment of IVC atresia. Both open surgical reconstruction via bypass and endovascular reconstruction are used for the management of this condition [9]. As of now, there is limited evidence to support either an open or endovascular approach for the management of IVC atresia. Because of the low incidence of IVC atresia, and limited reported surgical experience, any conclusion might be far-fetched [4]. However, these options should be selectively offered to a limited subset of IVC atresia patients with advanced chronic insufficiency as high failure rates requiring reintervention have been reported with both open and endovascular treatment [10,11].

## Conclusions

IVC atresia is a rare vascular anomaly but a cause of recurrent DVTs and venous ulcers. Patients affected are usually young and suffer from recurrent bilateral DVTs. CT and MRI investigations are useful in diagnosis and should be ordered as part of further workups for unprovoked DVTs. Lifelong anticoagulation is generally advised for frequent DVTs; however, catheter-directed thrombolysis as an adjunct is also utilized. Surgical options to cure IVC atresia are less studied and often not offered due to the rarity of the condition.
